# Chronic wasting disease (CWD) prion strains evolve via adaptive diversification of conformers in hosts expressing prion protein polymorphisms

**DOI:** 10.1074/jbc.RA120.012546

**Published:** 2020-02-28

**Authors:** Camilo Duque Velásquez, Chae Kim, Tracy Haldiman, Chiye Kim, Allen Herbst, Judd Aiken, Jiri G. Safar, Debbie McKenzie

**Affiliations:** ‡ Department of Biological Sciences, University of Alberta, Edmonton, Alberta T6G 2R3, Canada; ¶ Department of Pathology, Case Western Reserve University, Cleveland, Ohio 44106; ** Department of Neurology, Case Western Reserve University, Cleveland, Ohio 44106; ‖ Department of Agricultural, Food and Nutritional Sciences, University of Alberta, Edmonton, Alberta T6G 2P5, Canada; § Centre for Prions and Protein Folding Diseases, University of Alberta, Edmonton, Alberta T6G 2M8, Canada

**Keywords:** prion, genetic polymorphism, conformational change, oligomer, prion disease, host–pathogen interaction, evolution, host range, strains

## Abstract

Chronic wasting disease (CWD) is caused by an unknown spectrum of prions and has become enzootic in populations of cervid species that express cellular prion protein (PrP^C^) molecules varying in amino acid composition. These PrP^C^ polymorphisms can affect prion transmission, disease progression, neuropathology, and emergence of new prion strains, but the mechanistic steps in prion evolution are not understood. Here, using conformation-dependent immunoassay, conformation stability assay, and protein-misfolding cyclic amplification, we monitored the conformational and phenotypic characteristics of CWD prions passaged through deer and transgenic mice expressing different cervid PrP^C^ polymorphisms. We observed that transmission through hosts with distinct PrP^C^ sequences diversifies the PrP^CWD^ conformations and causes a shift toward oligomers with defined structural organization, replication rate, and host range. When passaged in host environments that restrict prion replication, distinct co-existing PrP^CWD^ conformers underwent competitive selection, stabilizing a new prion strain. Nonadaptive conformers exhibited unstable replication and accumulated only to low levels. These results suggest a continuously evolving diversity of CWD conformers and imply a critical interplay between CWD prion plasticity and PrP^C^ polymorphisms during prion strain evolution.

## Introduction

Prions are infectious agents composed of misfolded, templating conformations (*e.g.* scrapie prion protein (PrP^Sc^))^2^ of host (*Prnp*-encoded) cellular prion proteins (PrP^C^) (1). Prion propagation and accumulation in brain cause chronic wasting disease (CWD) in cervids, cause scrapie and bovine spongiform encephalopathy, and in humans cause Creutzfeldt-Jakob disease (1). The products of prion replication comprise a range of PrP^Sc^ multimers with increased detergent insolubility and resistance to both protease digestion (*e.g.* proteinase K (PK)) and protein denaturants (*e.g.* GdnHCl) (2–5). Prion strains have been operationally defined by the heritability of specific disease phenotypes upon passage in hosts isogenic for PrP^C^ (6–8). In accordance with the prion hypothesis, these phenotypic changes have been ascribed to conformational and quaternary properties of PrP^Sc^ molecules (2–5, 7, 8).

Prion strains can adapt when transferred between host species expressing different PrP^C^ molecules or between hosts and *in vitro* cell models (6, 8–10). Strain emergence can occur following transmission to novel host–PrP^C^ environments (6, 8, 9); the mechanism behind this adaptive response, however, remains unclear (9, 10). Prion conformers have been postulated to “mutate” by deformed-templating (11). We hypothesize a prion strain is a conformational species that diversifies during replication in novel host PrP^C^ environments that limit the propagation of a colonizing lineage, leading to the emergence of novel strains.

The spread of CWD is promoted by host–to–host interactions and environmental exposure to persistent prion infectivity released in biofluids and carcasses of diseased cervids (12–14). Susceptibility to prion disease is determined by route of exposure, infectious dose, the host PrP^C^, and the invading prion strain (15–18). CWD is transmitted between cervids expressing different PrP^C^ polymorphisms, which can modulate disease progression, strain selection, and pathogenicity (15, 19–24). Thus, whereas cervids expressing specific PrP^C^ molecules succumb faster when infected with a specific strain, those expressing PrP^C^ polymorphisms can have extended incubation periods (15, 22, 24). PrP^C^ variation at residues 95 (Gln/His) and 96 (Gly/Ser; most common PrP^C^ allelotypes) affects susceptibility of white-tailed deer (*Odocoileus virginanus*) to CWD (15, 19–22). The protective effect of the Ser-96–PrP^C^ transmission barrier has been implicated in the increased frequency of Ser-96–*PRNP* alleles in deer populations chronically exposed to CWD (23).

To determine the effects of nonhomologous prion replication, we measured the biochemical, biophysical, and infectious properties of CWD prions generated in white-tailed deer expressing combinations (*i.e.* allotypes) of His-95, Ser-96, or Gln-95/Gly-96 (WT) PrP^C^. Although all deer were dosed with the same prion isolate (Wisc-1), deer expressing His-95–PrP^C^ accumulated a mixture of an emergent strain H95^+^ and invading Wisc-1. Here, we demonstrate the conformational diversification of cervid prions during replication in nonhomologous host–PrP^C^ environments and how that diversification leads to the emergence of a new CWD strain.

## Results

### Novel PrP^CWD^ properties in deer expressing PrP^C^ polymorphisms

**Figure 1.**
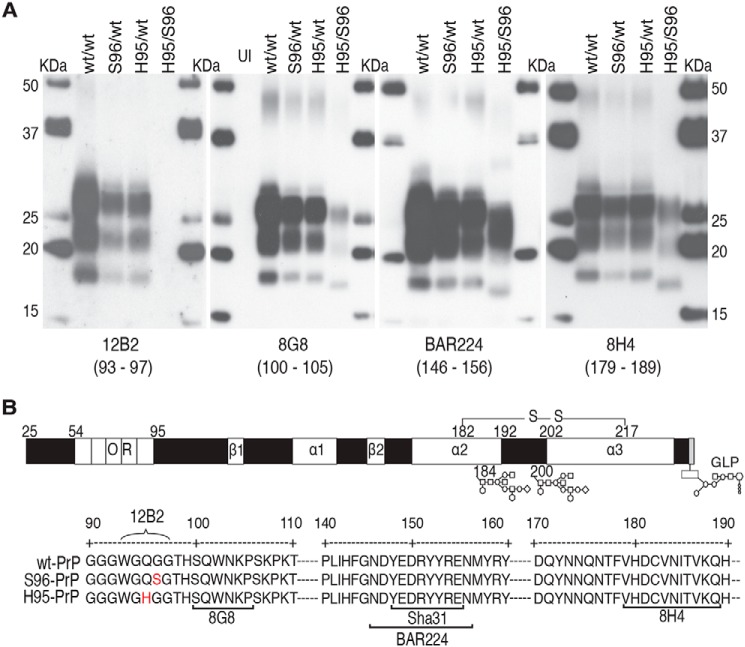
**Novel PrP^CWD^ properties following Wisc-1 propagation in deer expressing PrP^C^ polymorphisms.** PrP^CWD^ profiles were obtained from whole hemi-encephalon homogenates derived from orally-infected white-tailed deer expressing different PrP^C^ primary structures (*PRNP* alleles). *A,* PK-resistant (res) PrP^CWD^ signatures detected with monoclonal antibodies (mAbs) 12B2 (deer; 93–97), 8G8 (100–105), BAR224 (146–156), and 8H4 (179–189). Expression of histidine or serine at residues 95 and 96, respectively, disrupted 12B2 detection of these PrP^CWD^ allelotypes. For other mAbs, detection of His-95/Ser-96 PK-res PrP^CWD^ required three times more brain protein equivalents. *UI,* uninfected control. Previously described detection with 8G8 mAb (15) was included for the purpose of comparison. *B,* linear representation and partial primary sequence of deer PrP^C^ displaying amino acid polymorphisms (*red*), secondary structural motifs (*white boxes*: β, β-sheets; α, α-helices), and the epitopes of mAb used for detection; Sha31(148–155). Residues 184 and 200 indicate variable *N*-linked glycosylation residues; *GLP*, glycolipid.

Replication of a prion isolate, from hunter-harvested WT/WT white-tailed deer (passage 0 (P0)), in deer expressing PrP^C^ polymorphisms resulted in PrP^CWD^ (cervid PrP^Sc^) with different glycotype patterns and epitope binding (Fig. 1, *A* and *B*). The His-95/Ser-96 PrP^CWD^ exhibited distinct PK cleavage profiles and antibody reactivity compared with His-95/WT, Ser-96/WT, and WT/WT PrP^CWD^, which contained PrP with similar protease resistance (Fig. S1). The PrP^CWD^ in four additional WT/WT and Ser-96/WT deer from the initial propagation of the Wisc-1 isolate (15) were similar to each other but were also distinct from the His-95/Ser-96 PrP^CWD^ (data not shown).

**Figure 2.**
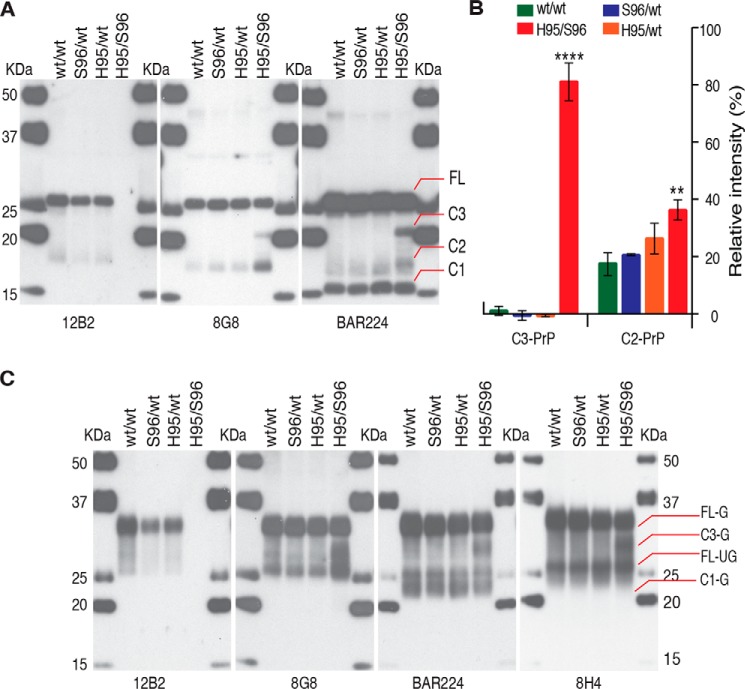
**Alternative N-terminal endoproteolytic processing and antibody reactivity of total PrP in brain of CWD-infected deer expressing different PrP^C^ allelotypes.** *A,* de-glycosylated total PrP in Wisc-1–infected white-tailed deer expressing WT-(Q95G96)–PrP^C^, Ser-96–PrP^C^, or His-95–PrP^C^ allelotypes. His-95/Ser-96 deer accumulated a distinctive N-terminally–cleaved PrP (*C3*) of ∼20 kDa following removal of glycans with PNGase F. Full-length (*FL*) PrP, C-terminal PrP (*C1, C2,* and *C3*). *B,* percentage of de-glycosylated C3- and C2–PrP detected with BAR224. Mean with standard deviation is indicated by *bars*. Densitometry was performed in three separate de-glycosylation experiments for each individual deer CWD lineage. The brain of His-95/Ser-96 deer contained significantly higher levels of C3- and C2–PrP than other deer. ANOVA, *p* < 0.05. Differences in C2–PrP abundance were 4–5-fold larger with 8G8 (data not shown). *C*, total deer brain PrP (*G*, glycosylated; *UG,* unglycosylated). The mAb 12B2 (epitope WGQGG, deer residues 93–97) does not recognize Ser-96–PrP^C^ or His-95–PrP^C^. The abundance of full-length and C1-PrP between deer was equivalent ruling out post-homogenization degradation.

Alternative N-terminal truncation was observed following de-glycosylation of total PrP (PrP^C^ + PrP^CWD^), with the His-95/Ser-96 deer brain homogenate displaying a distinct C-terminal PrP fragment (C3–PrP) and higher levels of C2–PrP with 8G8 or BAR224 mAb (Fig. 2, *A* and *B*). In the His-95/Ser-96 deer brain, this C3–PrP existed in an abundantly glycosylated state with an apparent mass of 30 kDa prior to glycan removal (Fig. 2*C*). Both polymorphisms occur within the ^93^WGQGG^97^ epitope of the 12B2 mAb, preventing detection of both His-95 and Ser-96 PrP^CWD^ and PrP^C^ (Figs. 1*A* and 2*C*).

Using europium-labeled mAb 12B2 (epitope 89–93) and 8G8 (epitope 97–102) in the conformation-dependent immunoassay (CDI), the concentration of cervid PrP^C^ was measured directly in a native untreated aliquot of the sample after calibration with recombinant monomers of α-helical prion protein (Table 1). Previously, CDI monitoring of PrP^C^ in seven rodent-adapted prion strains, including rodent-adapted CWD, revealed a major down-regulation (26–28). A second aliquot of the same sample was denatured with 4 m GdnHCl, and the increase in the time-resolved fluorescence signal due to the newly-exposed epitopes over that recorded in native state is proportional to the concentration of PrP^CWD^ (2–5, 29–32). If this step is performed before and after PK treatment, subtracting the concentration of the protease-resistant core (PK-res PrP^CWD^) from total PrP^CWD^ in an untreated sample determines a concentration of soluble protease-sensitive prion conformers (PK-sen PrP^CWD^) (2–5, 32). We have shown previously that the levels of prion oligomers in distinct prion strains and differential protease resistance are important differentiating characteristics of rodent strains (2, 26, 29, 33–34) and human prion strains (3–5, 30, 32, 35, 36). Using CDI with 12B2, we measured the abundance of WT–PrP in homozygous and heterozygous deer. At clinical stages, the whole hemi-encephalon homogenates of Ser-96/WT and His-95/WT deer contained less total PK-sensitive and PK-resistant PrP^CWD^ than WT/WT deer (Table 1). This decrease is less than expected based upon WT–PrP^C^ gene dosage. The calibration of CDI performed with Eu-12B2 and Eu-8G8 mAbs with full-length recombinant PrP(23–231) had a correlation coefficient close to 1 (Fig. S2*A*) and demonstrated equivalent concentrations of PrP in WT/WT deer brain homogenates (Fig. S2*B*). As outlined in Fig. 1, the 12B2 antibody cannot be used to measure accurately PrP with the His-95 or Ser-96 polymorphisms.

**Table 1:** **The CDI measurements of WT–PrP were performed with Eu-12B2 mAb and PrP with His-95 or Ser-96 polymorphisms with Eu-8G8 mAb**

CWD allotype	PrP^C^		Total PrP^CWD^		PK-res PrP^CWD^	
	*ng/ml*	*S.E.*	*ng/ml*	*S.E.*	*ng/ml*	*S.E.*
WT/WT–12B2	715.0	3.0	474.0	2.0	226.0	1.9
Ser-96/WT–12B2	592.0	7.0	149.0	0.9	74.0	0.3
His-95/WT–12B2	467.0	4.0	205.0	1.1	78.0	0.3
His-95/Ser-96–8G8	702.9	68.4	35.2	0.7	10.8	0.4

### Monitoring conformational diversification of CWD prions in deer expressing polymorphic PrP^C^ with conformational stability assay (CSA)

**Figure 3.**
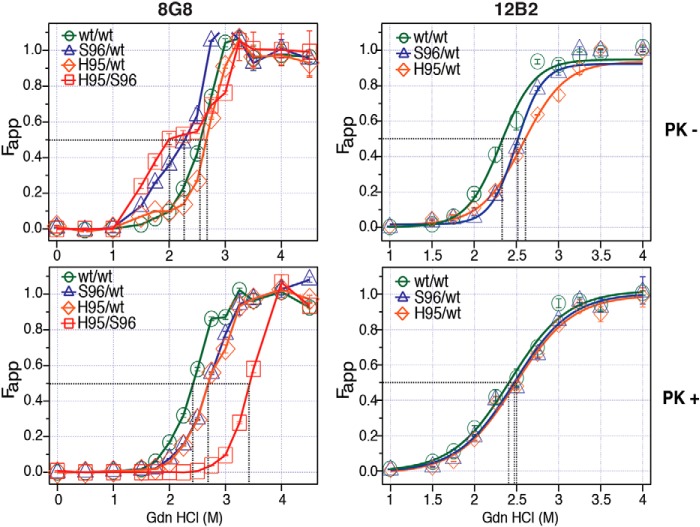
**Conformational diversification of Wisc-1 prions in deer expressing PrP^C^ polymorphisms Ser-96 and His-95.** Structural stability of deer PrP^CWD^ after exposure to increasing concentrations of GdnHCl (m). The apparent fractional change (*F*_app_) of unfolding was measured by CDI with 8G8 or 12B2 mAbs before or after proteinase K treatment (*PK* −/+). CWD conformers encoded in all three PrP primary structures were detected with 8G8 mAb, whereas 12B2 only recognized prions encoded on WT–PrP. *Black dotted lines* indicate the denaturation midpoints (GdnHCl_1/2_). *F*_app_ values and *bars* represent the mean ± S.E. obtained for each individual deer CWD lineage (two batches of 20% w/v brain homogenate) measured in triplicate with each Eu-labeled mAb.

The prion denaturation performed sequentially with incremental concentrations of GdnHCl in CSA allows comparison of protein stabilities of distinct prion strains due to the differences in the structural organization (2–5, 26, 28, 32–34, 37). The dissociation and unfolding of β-sheet structured aggregates of PrP^Sc^ in the presence of incremental concentrations of GdnHCl has been previously described as follows: [PrP^Sc^]n → [PK-sen PrP^Sc^]n → iPrP → uPrP, where [PrP^Sc^]n indicates native (n) aggregates of PrP^Sc^; [PK-sen PrP^Sc^]n indicates soluble protease-sensitive oligomers of PrP^Sc^; iPrP is an intermediate; and uPrP is completely unfolded (denatured) PrP (38–40). Using CSA, we monitored the transition from [PrP^CWD^]n to completely unfolded uPrP, and we used either 8G8 or 12B2 mAbs to compare the GdnHCl resistance of WT, His-95, and Ser-96 PrP^CWD^ generated by Wisc-1 homologous and nonhomologous PrP^C^ conversion (Fig. 3). CWD conformers encoded in the three PrP allelotypes were detected with 8G8 mAb, whereas 12B2 only recognizes WT–PrP^CWD^ conformers. Without PK(−) treatment, WT/WT PrP^CWD^ unfolded in a single phase with GdnHCl_1/2_ between 2.3 and 2.5 m depending on the detection antibody (Fig. 3, *top panels*). In contrast, in the Ser-96/WT deer, we observed lower stability PrP^CWD^ multimers, whose unfolding was characterized by a 2–3-fold larger apparent fractional change of unfolding (*F*_app_) compared with WT/WT PrP^CWD^ at GdnHCl concentrations between 1.5 and 2.2 m (Fig. 3, *top left panel*). These low stability multimers were not observed with the 12B2 antibody indicating they are composed of Ser-96–PrP^CWD^ (Fig. 3, *top right panel*). In contrast, the His-95/WT total PrP^CWD^ repertoire had higher denaturation midpoints (2.6–2.7 m) with both mAbs compared with the unfolding properties of Wisc-1 PrP^CWD^ on WT/WT deer. The His-95/WT total PrP^CWD^ spectrum was the most resistant to GdnHCl unfolding of the mAb N-terminal epitopes. Cumulatively, the His-95/Ser-96 deer brain contained higher PrP^CWD^ conformational diversity, including the low-stability multimers and quaternary arrangements with high stability compared with WT/WT PrP^CWD^ at 2.7 and 3.0 m GdnHCl (Fig. 3, *top left panel*).

Protease treatment (PK+) removed the low-stability Ser-96 PrP^CWD^ detected by 8G8 in the Ser-96/WT and His-95/Ser-96 CWD allotypes as indicated by the single-phase denaturation curves (Fig. 3, *bottom left panel*). Unlike the GdnHCl_1/2_ (m) of the His-95/WT PrP^CWD^ (measured with the 8G8), all denaturation midpoints shifted following PK digestion. The GdnHCl_1/2_ (m) of all PK-res PrP^CWD^ combinations ranged from 2.4 to 3.4 m. PK-res Wisc-1 prions in WT/WT deer unfolded at lower GdnHCl concentrations than the products of nonhomologous conversion in Ser-96/WT and His-95/WT CWD allotypes (2.4 < 2.7 m). When, however, CSA was performed with the 12B2 mAb, the GdnHCl_1/2_ (m) of WT–PrP^CWD^ in Ser-96/WT and His-95/WT deer differed from WT/WT deer by ∼0.1 m suggesting that these conformers are identical (Fig. 3, *bottom right panel*). The propensity of PK-res PrP^CWD^ to unfold and expose the corresponding mAb epitopes was increased in deer carrying more conformationally-diverse prions compared with Wisc-1 (generated by homologous conversion in WT/WT deer). Overall, the His-95 and Ser-96 PrP^CWD^ combinations were more resistant to GdnHCl denaturation than WT–PrP^CWD^. These unfolding spectra of total (PK−) and PK-res PrP^CWD^ demonstrate alternative prion conformers emerged in heterozygous deer.

**Figure 4.**
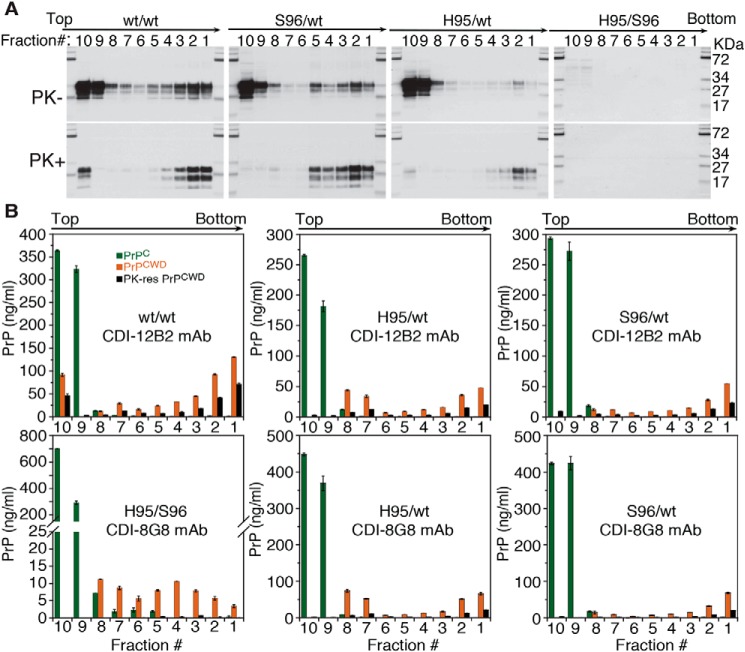
**Sedimentation properties and protease resistance of PrP^CWD^ from Wisc-1 deer CWD lineages.** Fractions collected from the bottom of the tube were analyzed by Western blotting and CDI prior (−) or after (+) treatment with proteinase K. *A,* distribution of total and PK-res WT–PrP^CWD^ in different CWD allotypes detected with 12B2 mAb. *B,* concentration of fractionated WT–PrP^C^ (*green*), total WT–PrP^CWD^ (*orange*), and PK-res WT–PrP^CWD^ (*black*) was determined for WT/WT, Ser-96/WT, and His-95/WT prions using CDI with 12B2 mAb. Detection of His-95 and Ser-96 PrP^CWD^ required 8G8 mAb. *Bars* represent average ± S.E. CDI measurements were performed on each individual deer sample in triplicate.

The properties of CWD prion multimers were further characterized by sedimentation velocity in sucrose gradients to compare the effects of His-95 and Ser-96 polymorphisms on the PrP^CWD^ multimerization states generated by Wisc-1 conversion in heterozygous deer (Fig. 4). The WT–PrP^CWD^ from homozygous and heterozygous deer had different sedimentation and protease resistance properties resolved by Western blotting (Fig. 4*A*) and measured by CDI with Eu-12B2 and Eu-8G8 mAbs (Fig. 4*B*). Compared with heterozygous deer, the WT/WT deer brain contained more PK-sen and PK-res PrP^CWD^ multimers at the top sucrose fraction (Fig. 4*B, top left panel*). The gradient distribution of WT–PrP^CWD^ in the His-95/WT deer brain was shifted toward more abundant small protease-sensitive oligomers that eluted in fractions 7–8 (Fig. 4*B, top center panel*). The sucrose gradient sedimentation profiles of PrP^CWD^ in Ser-96/WT deer with 12B2 and 8G8 mAbs were indistinguishable suggesting that PrP^CWD^ detected is primarily composed of WT–PrP (Fig. 4*B, right panels*). Interestingly, the concentration of PrP^CWD^ in the sucrose gradients as measured with Eu-8G8 antibody in His-95/WT deer was 2-fold higher than the concentration obtained with Eu-12B2 suggesting these prions are a mixture of WT and His-95 PrP^CWD^ (Fig. 4*B, bottom center panel*). However, the definite proof will require purification of PrP^CWD^ and sequencing by MS. The PrP^CWD^ multimers accumulated in the His-95/Ser-96 deer brain were primarily composed of small protease-sensitive oligomeric species, whereas the PK-res PrP^CWD^ components were detected in the bottom fractions 3–5 (Fig. 4*B, bottom left panel*).

### Differential host selection of strain-specific CWD conformers

**Figure 5.**
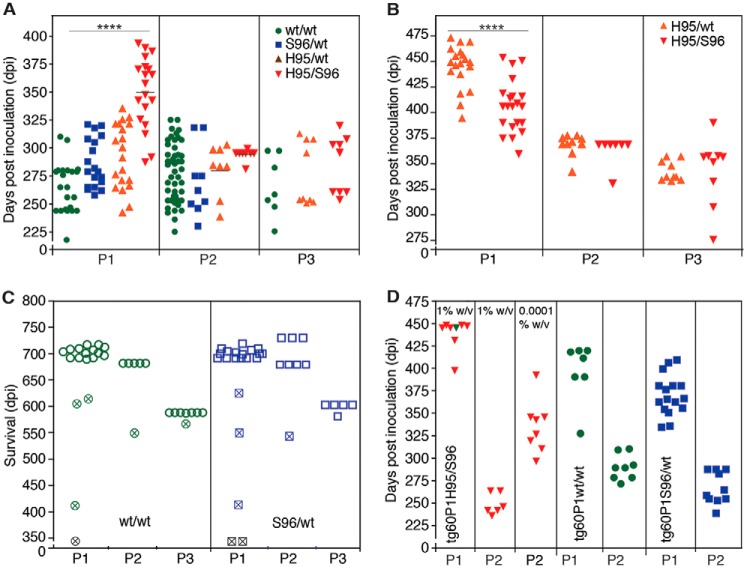
**Selection, adaptation, and unstable propagation of CWD prion lineages in tg33 and tg60 mice expressing deer G96 (WT) and Ser-96–PrP^C^, respectively.** Unless otherwise noted, all inocula were 10% w/v brain homogenates. *A,* incubation periods for tg33 mice infected with WT/WT (*green*), Ser-96/WT (*blue*), His-95/WT (*orange*), and His-95/Ser-96 (*red*) CWD and their descendent prion conformations. Incubation periods were shortened during serial passage (*P1–P3*) (****, *p* < 0.0001; Kruskal-Wallis, Dunn's multiple comparison test). *B,* rapid adaptation of a prion species (H95^+^) descending from Wisc-1 infection in His-95/WT and His-95/Ser-96 deer during passage in tg60 mice (****, *p* < 0.0001; Mann-Whitney test). *C,* unstable propagation of WT/WT and Ser-96/WT prions in tg60 mice failed to produce clinical prion disease. *Open symbols* represent mice that survived until the end of the experiment. *Crossed symbols* represent mice with intercurrent disease or time points for PrP^CWD^ evaluation and subsequent passage. *D,* comparison of prion doses accumulated in tg60 mice by bioassay in tg33 mice. P1 inocula are indicated and were from tg60 mice euthanized after 359 (*red*; shown in *B*) and 345 dpi (*crossed symbols*; shown in *C*). Stable H95^+^ prion replication with Ser-96–PrP^C^ generated ∼10-fold more prions than WT/WT and Ser-96/WT prion species. Some transmission data (*P1*, except for *D*, and *P2* for the tg60 in *B*) were previously reported (24) and are reproduced here for comparison. A single modification was made to the reproduced data (*C*; time points 345 days post inoculation were not previously reported). The P2 in tg60 mice in *B* was also reproduced from Ref. 24.

**Figure 6.**
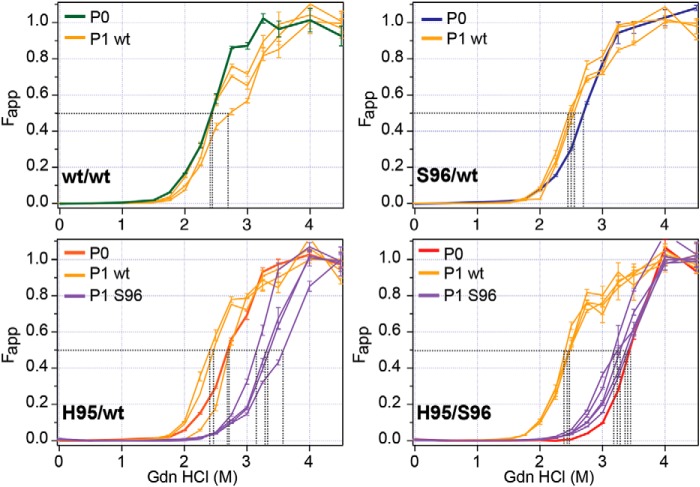
**Selection of strain-specific prion conformers in transgenic mice expressing deer PrP^C^ polymorphisms.** Conformational stability of different CWD lineages propagated (*i.e.* encoded) in Gly-96 (WT) or Ser-96–PrP from transgenic mice. *P0* refers to the unfolding of Wisc-1 replication products after homologous prion conversion in WT/WT deer and heterologous conversion in Ser-96/WT, His-95/WT, and His-95/Ser-96 deer. P1 describes the GdnHCl unfolding resistance of PrP^CWD^ conformational descendents stably amplified after first passages in tg33 (WT; *yellow*) and tg60 (Ser-96; *purple*) mice. The apparent fractional change (*F*_app_) of unfolding was determined by CDI with 8G8 mAb after proteinase K treatment. *Black dotted lines* indicate the denaturation midpoints (GdnHCl_1/2_). The H95^+^ strain conformers differed in their adaptability in mice expressing serine instead of glycine at amino acid 96. PK-res PrP^CWD^ was either absent or below detection levels in WT/WT and Ser-96/WT infected tg60 mice. *F*_app_ values and *bars* represent the mean ± S.E. measured in 10% w/v brain homogenates from three to five prion disease affected mice and measured in triplicate with Eu-8G8-labeled mAb.

**Figure 7.**
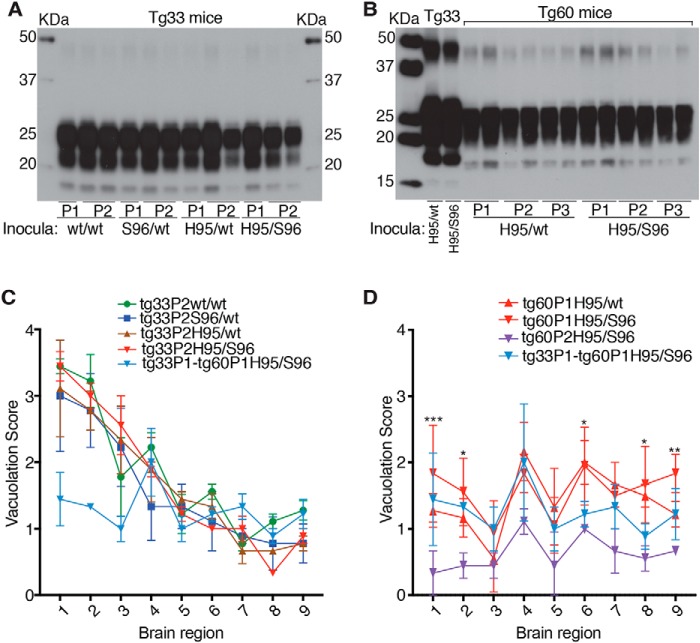
**Stable strain biochemical and neuropathological phenotypes after serial passage of CWD lineages in tg33 and tg60 mice.** *A,* PK-res PrP^CWD^ in groups of tg33 from first (*P1*) and second (*P2*) passages. *B,* PK-res PrP^CWD^ in tg60 (Ser-96–PrP^C^) mice across three passages (*P1–P3*). *C,* similar lesion profiles in sagittal brain sections from tg33 mice following P2. Score values represent the mean ± S.D. (two-way ANOVA; *, *p* < 0.05). Lesion profile of tg33 mice infected with tg60P1His-95/Ser-96 prions (*light blue*) is included for comparison. *D*, vacuolation profiles in sagittal brain sections from different passages (*P1* and *P2*) of clinically affected tg60 mice infected with H95^+^ strain. The degree of vacuolation was significantly lower after the second passage of the His-95/Ser-96 lineage (***, *p* < 0.0001; **, *p* < 0.001; *, *p* < 0.01 two-way ANOVA). Lesion profile of tg33 mice infected with tg60P1His-95/Ser-96 prions (*light blue*) was not included in the statistical comparison. The H95^+^ strain brain region targeting was similar in tg33 and tg60 mice. *Brain regions: 1,* cerebral cortex; *2,* cerebral nuclei; *3,* hippocampus; *4,* thalamus; *5,* hypothalamus; *6,* midbrain; *7,* pons; *8,* medulla; and *9,* cerebellum. Western blot analysis was performed in 3–6 mice from each passage line (not all included in *A*). Lesion profile analysis and statistical comparison (in *D*) was performed in groups of three animals per passage line.

**Figure 8.**
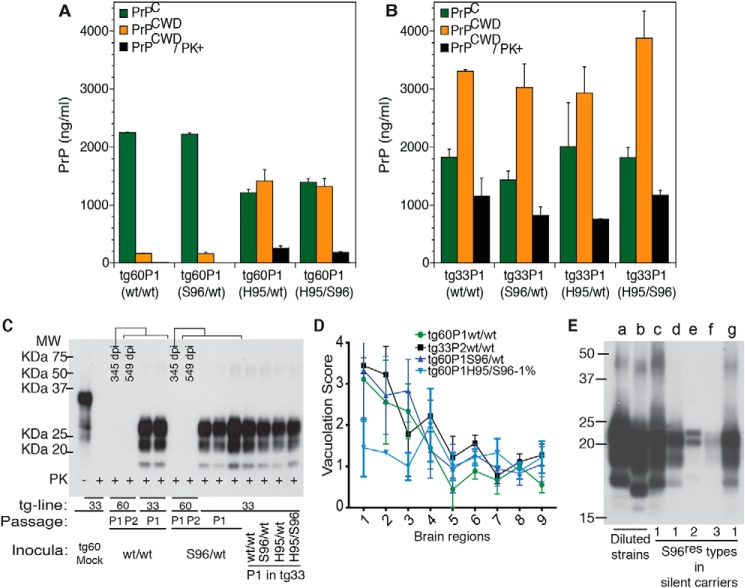
**PrP^CWD^ abundance in CWD-infected transgenic mice and phenotypic stability after allogenic prion passage.** The average levels of PrP^C^, total PrP^CWD^, and PK-res PrP^CWD^ were measured by CDI with 8G8 mAb. *A,* Tg60 mice exposed to WT/WT (*i.e.* Wisc-1) and Ser-96/WT deer prions accumulated low levels of PK-sen PrP^CWD^ compared with mice receiving His-95/WT and His-95/Ser-96 (*i.e.* H95^+^) prions. *B,* Tg33 mice accumulated similar levels of PrP^CWD^. *C,* host replication of PK-sen S96 PrP^CWD^ in tg33 mice (Fig. 5*D*) (Bar224 1:10.000). *D,* lesion profiles in sagittal brain sections from tg33 mice infected with tg60P1 prions. Score values represent the mean ± S.E. (two-way ANOVA; *, *p* < 0.05). Lesion profile of tg33P2WT/WT (*black*) is included for comparison. The lesion profile of tg33 mice infected with tg60P1His-95/Ser-96 prions (*light blue*) was not included in the statistical comparison. The H95^+^ strain brain region targeting was similar in tg33 and tg60 mice. *Brain regions: 1,* cerebral cortex; *2,* cerebral nuclei; *3,* hippocampus; *4,* thalamus; *5,* hypothalamus; *6,* midbrain; *7,* pons; *8,* medulla; and *9.* cerebellum. *E,* low abundance of novel S96^res^ types following first passage of WT/WT and Ser-96/WT CWD into individual tg60 mice in the absence of prion disease signs. Brains of tg-mice infected with Wisc-1 and H95^+^ (*a* and *b*), respectively, contained more PrP^CWD^ than asymptomatic tg60 carriers (*c–g*) as measured by Western blotting. Wisc-1 and H95^+^ strains were diluted 100- and 10-fold, respectively, in normal tg60 brain homogenate prior parallel PK treatment. Unlike standard detection conditions, a range between 240 and 300 μg (3.4–4.2 μg/μl) of brain total protein equivalents was incubated with 50–100 μg/ml of PK (70 μl reaction volume) to enhance detection. Samples (≈25–31 μg of protein equivalents-post PK) were resolved per well (Sha31 mAb 1:30,000). CDI analysis was performed in 3–5 mice per passage line with the exception of asymptomatic tg60P1(two mice). Histological analysis and statistical comparison were performed in groups of three mice per passage line.

**Figure 9.**
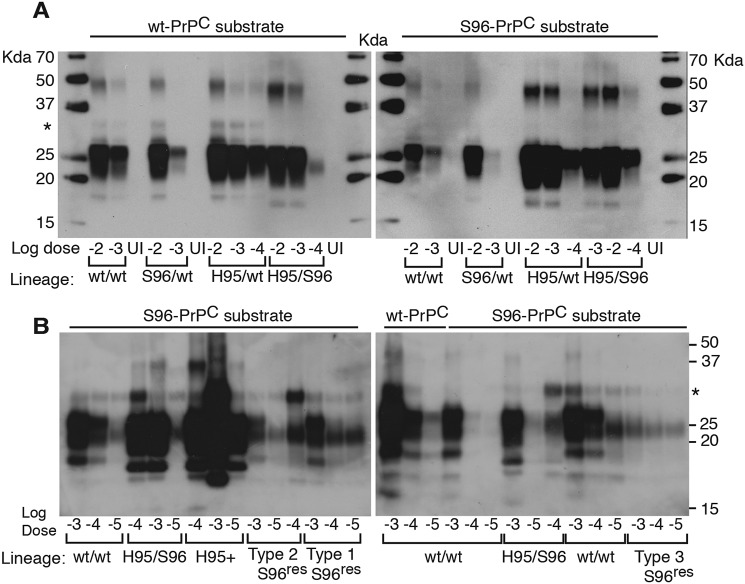
***In vitro* amplification of CWD lineages from deer and tg60 mice.** *A,* PMCA propagation of CWD lineages on WT–PrP and Ser-96–PrP^C^ substrates from tg33 and tg60 brains. Reactions in WT–PrP^C^ substrate seeded with WT/WT, Ser-96/WT, and His-95/WT prions amplified PrP-res pattern consistent with the Wisc-1 strain. *In vitro* conversion of Ser-96–PrP^C^ produced the Wisc-1 pattern when seeded with WT/WT and Ser-96/WT prions and the H95^+^ pattern when seeding was done with His-95/WT and His-95/Ser-96 CWD lineages. Accumulation of a high-molecular mass (30 kDa) PK-res PrP was also observed (*asterisk*). Amplification was a single 24-h round of PMCA. Uninfected control (*UI*) was seeded at 10^−2^. *B*, unstable replication of S96^res^ PrP^CWD^ types 1 and 2 during a single 48-h round of PMCA (*left panel*). Type 3 S96^res^ was amplified unaltered (*right panel*). PMCA of Wisc-1 (WT/WT) prions on Ser-96–PrP also produced type-3 PrP^CWD^ when seeded at larger dilutions. His-95/Ser-96 and tg60P1His-95/Ser-96 prions (*i.e.* H95^+^) were more stable generating the low molecular weight PK-res PrP^CWD^ characteristic of this strain. Each *panel s*hows PMCA reactions seeded at the same time and amplified in separate sonicators. Amplification conditions: 30 s sonication at 60% amplitude every 15 min at 37 °C. Western blotting detection of PrP-res was achieved with BAR224 (1:10,000) and Sha31 (1:30,000) mAbs (*A* and *B*, respectively).

**Figure 10.**
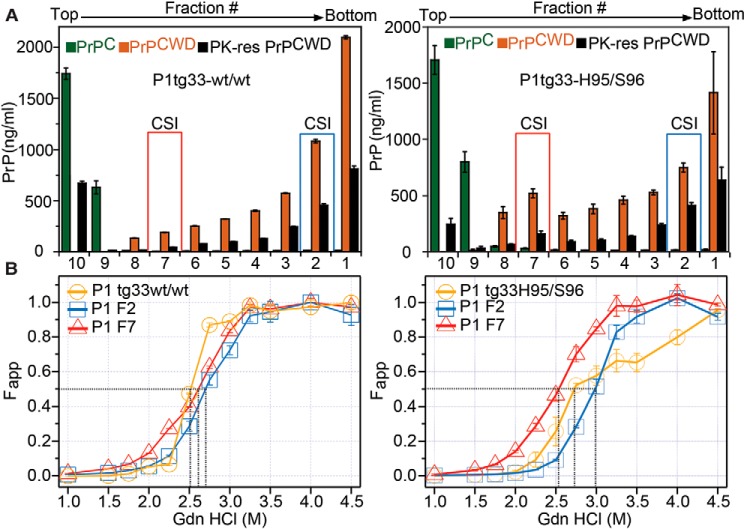
**Co-existence of Wisc-1 and H95^+^ conformers encoded in WT–PrP from tg33 mice.** Structural properties of WT–PrP^CWD^ in tg33 mice were exposed to Wisc-1 CWD (WT/WT) or the His-95/Ser-96 (a mixture of Wisc-1 and emergent H95^+^ strains) are shown. *A,* sedimentation velocity profiles. The concentration of fractionated WT–PrP^C^ (*green*), total WT–PrP^CWD^ (*orange*), and PK-res PrP^CWD^ (*black*) was determined using CDI with 8G8 mAb. *Bars* represent mean ± S.E. measured for 3–4 mice by triplicate. *B,* average structural stability (*yellow*) of total PrP^CWD^ (PK−) measured for groups of tg33 mice. Brain homogenates from tg33 mice contained PrP^CWD^ conformers with distinct structural stability separated by sedimentation velocity (*bar 7, red,* and *bar 2, blue*). The apparent fractional change (*F*_app_) of unfolding was determined by CDI using 8G8 mAb. *Black dotted lines* indicate the denaturation midpoints (GdnHCl_1/2_ m). *Bars* represent mean ± S.E. Sedimentation and conformation analysis were performed in 3–5 mice per passage line.

To determine whether PrP^CWD^ conformations emerging in heterozygous deer were reproducible upon passage, we inoculated the four (P0) Wisc-1 CWD lineages into differentially susceptible tg33 (WT–PrP^C^) and tg60 (Ser-96–PrP^C^) mice, resulting in the propagation and isolation of distinct prion species (Fig. 5, *A–C*). The biochemical signatures, neuropathology, structural and transmission properties, as well as the infectivity levels associated with the four PrP^CWD^ lineages (P0) and their descendants (P1 to P3) were ascertained by strain identity (Figs. 5–10 and Table 2).

Following first passage (P1) of deer prions, the unfolding of PK-res PrP^CWD^, amplified in tg33 mice, had GdnHCl_1/2_ values ranging between 2.4 and 2.7 m as determined by CSA with 8G8 mAb (Fig. 6, *yellow*). This range covers the structural-resistance spectra of WT/WT, His-95/WT, and Ser-96/WT PrP^CWD^ (Fig. 6, *P0*). Conversely, PrP^CWD^ accumulated in clinically-affected tg60 mice displayed higher resistance to unfold (GdnHCl_1/2_ > 3.0 m identical to the structural stability and unfolding spectra of the PK-res His-95/Ser-96 PrP^CWD^) (Fig. 6, *purple* and *red*). PrP^CWD^ from tg60 mice exposed to His-95/WT CWD had similar denaturation midpoints (GdnHCl_1/2_ > 3.0 m) as the His-95/Ser-96 prions (Fig. 6, *purple*).

Serial propagation of these CWD lineages resulted in reduction of incubation periods (Fig. 5, *A* and *B*) with reproducible PrP^CWD^ glycotype pattern, vacuolation profiles (Fig. 7), and a reduction of conformational heterogeneity compared with the founding P0 lineages (see Figs. 3 and 6). Together, these data indicate that tg33 mice favored selection and replication of PrP^CWD^ conformers encoding a similar strain (*i.e.* Wisc-1), whereas passage in tg60 mice selected conformers encoding the H95^+^ strain.

Susceptibility of tg mice to distinct prion species (*i.e.* Wisc-1 or H95^+^) depended on the ability of either strain to convert PrP^C^ into PrP^CWD^ as compared by bioassay (Fig. 5*D*). Inoculation of 10-fold different brain homogenate doses of first passage tg60 prions (shown in Fig. 5, *B* and *C*) into tg33 mice resulted in comparatively similar incubation periods suggesting differences in accumulated titer (Fig. 5*D*). These differences in PrP^CWD^ dose were confirmed by CDI measurement of PrP^CWD^ in the brain of tg60 mice (Fig. 8*A*). Wisc-1–exposed tg60 mice accumulated low levels of PrP^CWD^ (∼7-fold less than His-95^+^–infected mice). This PrP^CWD^ resulted in clinical disease when passaged into tg33 mice producing PrP^CWD^ glycotypes and vacuolation profiles consistent with Wisc-1 infection (Fig. 8, *C* and *D*).

Western blot analysis of brain tissues of WT/WT and Ser-96/WT–inoculated tg60 mice (asymptomatic after >500 dpi) identified at least three types of PK-res Ser-96–PrP (S96^res^) (Fig. 8*E*). Based on semi-quantitative Western blot analysis, these Ser-96 PK-res PrP types are at least 10-fold less abundant than H95^+^ prions in clinically affected tg60 mice and 100-fold less abundant than Wisc-1 prions in tg33 mice. Detection of novel S96^res^ types required digestion of larger amounts of brain–protein equivalents (>200 μg) compared with standard PK digestion conditions (80–100 μg of total protein) (Fig. 8*E*). Blind passage of brain homogenates from asymptomatic tg60 mice did not lead to strain adaptation when inoculated into tg60 mice and resulted in re-isolation of Wisc-1 when transmitted into tg33 mice (Figs. 5, *C* and *D,* and 8, *C* and *D*).

### Unstable in vitro amplification of Wisc-1 prions and S96^res^ types with Ser-96–PrP^C^

To evaluate the replication properties of CWD lineages in tg60 mice, we used protein-misfolding cyclic amplification (PMCA) with WT or Ser-96–PrP^C^ as substrate (Fig. 9). Amplification of WT/WT and Ser-96/WT CWD in WT and Ser-96 PrP^C^ substrates resulted in a single profile consistent with propagation of the Wisc-1 strain (Fig. 9*A* and Fig. S3). In contrast, reactions seeded with the His-95/Ser-96 CWD lineage resulted in PrP-res indicative of the H95^+^ strain. Interestingly, His-95/WT prions produced the Wisc-1 pattern when amplified on WT–PrP^C^ substrate and H95^+^ when amplified on Ser-96–PrP^C^ (Fig. 9*A*). PrP^CWD^ conformers specifying these two strains exist in different ratios in the brains of His-95/Ser-96 and His-95/WT deer, as determined by bioassay and PMCA (Figs. 5–9 and Table 2).

**Table 2:** **Wisc-1 tg33-specific titers in CWD lineages generated in deer expressing PrP^C^ polymorphisms Ser-96 and His-95**

Dilution factor*^a^*	No. of positive mice/no. of mice inoculated*^b^* (incubation period range; days post-inoculation)			
−2	8/8 (306–354)	6/6 (302–349)	3/3 (332–378)	4/4 (350–414)
−3	8/8 (346–389)	6/6 (346–364)	7/7 (332–417)	4/4 (421–488)
−4	8/8 (375–427)	6/6 (381–485)	8/8 (354–458)	7/7 (533–663)
−5	8/8 (374–482)	6/6 (403–546)	9/9 (436–514)	3/12 (505–659)
−6	8/8 (391–550)	6/6 (462–578)	8/8 (555–633)	1/11 (673)
−7	4/8 (389–570)	1/10 (605)	4/6 (515–612)	1/8 (663)
Wisc-1 CWD lineages (P0)	WT/WT	S96/WT	H95/WT	H95/S96
Log i.c. ID_50_·μl^−1^	6.944	6.489	6.696	4.966
Log i.c. ID_50_·μl^−1^	8.4	8.0	8.2	6.4

*^a^* Serial 10-fold dilutions of 10% BH w/v.

*^b^* Intercurrent disease or found dead was not included.

PMCA analysis of WT/WT and Ser-96/WT deer and of asymptomatic tg60 mice infected with Wisc-1 demonstrated that the H95^+^ strain was not present (Fig. 9, *A* and *B*). Our PMCA assay was sensitive to a 10 million-fold dilution of Wisc-1 on WT–PrP substrate and 10,000-fold more sensitive than Western blotting (Fig. S3).

Most PMCA reactions shared a high molecular mass PK-res PrP^CWD^ fragment (∼30 kDa) that was propagated in either PrP^C^ substrate with different efficiencies and together with the Wisc-1 or H95^+^ PrP-res patterns (Fig. 9, *asterisks*). The 30-kDa peptide was amplified in separate PMCA reactions seeded with the His-95/Ser-96 lineage. Likewise, PMCA reactions seeded with Wisc-1 (from WT/WT deer) and the S96^res^ types also generated this peptide (Fig. 9*B, asterisk*).

*In vitro* replication of the S96^res^ types was unstable during PMCA using Ser-96–PrP^C^ substrate (Fig. 9*B*). When type-1 (Wisc-1 pattern on Ser-96–PrP; see Fig. 8*E* and Fig. S3*A*) and type-2 were amplified using Ser-96–PrP^C^ substrate, the Wisc-1 pattern emerged when seeded at a high dose (1000-fold dilution) and type-3 when at lower dose (100,000-fold) (Fig. 9*B, left panel*). No variation was observed when type-3 was amplified (Fig. 9*B, right panel*). When passaged at high dilution on Ser-96–PrP^C^ substrate, some PMCA reactions seeded with Wisc-1 (on WT/WT deer) also generated the type-3 S96^res^ (Fig. 9*B, right panel*). In contrast, the more adapted H95^+^ strain produced higher amounts of the characteristic low molecular weight PrP-res demonstrating *in vitro* selectivity of the assay with Ser-96–PrP^C^ (Fig. 9*B, left panel*).

### PrP^CWD^ variants persist across passages and emerge in selective hosts

To evaluate for co-amplification of alternative WT/WT PrP^CWD^ conformers (*i.e.* type-2 and -3), we further characterized the structural and transmission properties of the prions propagated in tg33 mice, a permissive host.

Sedimentation velocity fractionation in combination with CDI and CSA revealed differences in the average levels of PrP^CWD^ in top sucrose fractions (fraction #10 to #7) of tg33 mice infected with the WT/WT or His-95/Ser-96 lineages (Fig. 10*A*). Likewise, the average GdnHCl_1/2_ (m) of PK-sen PrP^CWD^ from tg33 (P1) mice was increased (GdnHCl_1/2_ > 2.5 m), and the PrP^CWD^ unfolded in two phases compared with WT/WT-inoculated mice, suggesting the structural diversity in the His-95/Ser-96 CWD lineage was amplified on WT–PrP (Fig. 10*B, yellow*). Independent of the CWD lineage inoculated, PrP^CWD^ in fraction #7 had average GdnHCl_1/2_ between 2.5 and 2.6 m (Fig. 10*B*). In contrast, prions in fraction #2 differed between lineages. Although Wisc-1 PrP^CWD^ in these fractions had an average GdnHCl_1/2_ of 2.7 m, the fractions containing the H95^+^ strain encoded in WT–PrP^CWD^ conformers were more stable at the same concentration (Fig. 10*B, light blue*).

**Figure 11.**
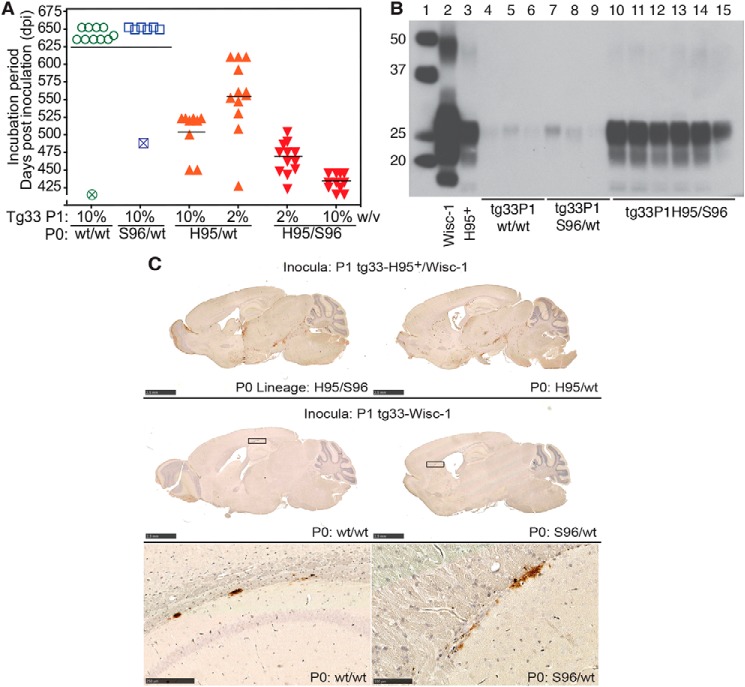
**Re-isolation of H95^+^ in tg60 mice inoculated with tg33 prions from different deer CWD lineages (*P0*).** *A*, selection of H95^+^ conformers in tg60 mice. Tg33 brain homogenates from individual mice of each primary transmission (P1) were inoculated into tg60 mice. *Open symbols* represent tg60 mice euthanized with no disease signs. *Crossed symbols* represent mice euthanized due to intercurrent disease. *B,* Western blotting of PK-res PrP^CWD^ in tg60 mice after exposure to tg33 mice prions. *Lane 1*, molecular weight marker. *Lane 2*, Wisc-1 from tg33 mice. *Lane 3,* H95^+^ from tg60 mice. *Lanes 4–6,* asymptomatic tg60 mice exposed to tg33-WT/WT prions, Wisc-1 strain. *Lanes 7–9,* asymptomatic tg60 mice exposed to tg33-Ser-96/WT prions, Wisc-1 strain. *Lanes 10–12,* tg60 mice exposed to tg33–His-95/WT prions. *Lanes 13–15,* tg60 mice exposed to tg33–His-95/Ser-96 prions, H95^+^/Wisc-1 strains mixture. *C,* PrP^CWD^ deposition pattern of H95^+^ strain in tg60 mice exposed to P1 tg33 brain prions consisting of co-propagated Wisc-1 and H95^+^ strains (*upper panel*). Distribution of PrP^CWD^ aggregates in tg60 mice exposed to prions from P1-tg33 mice that replicated the Wisc-1 strain (*middle* and *bottom panels*). PrP^CWD^ aggregates in between the corpus callosum and the hippocampus and in between the corpus callosum and the caudate putamen from in tg60 mice exposed to Wisc-1 from the WT/WT or Ser-96/WT lineages (*squares* in *middle panel*). All tissues were stained with BAR224 mAb. *Bars* (*upper* and *middle panels*), 2.5 mm. *Bar* (*bottom left panel*), 250 μm. *Bar* (*bottom right panel*), 100 μm. Western blotting and histological analyses were performed in 3–6 mice per passage line.

Given the adaptive properties of H95^+^ on hosts expressing WT or Ser-96–PrP^C^ (Fig. 5, *B* and *D*), we inoculated tg60 mice with P1 tg33 prions to evaluate for co-amplification of prion conformers. The incubation periods, neuropathological phenotypes, and PrP^CWD^ type of tg60 mice exposed to tg33–His-95/Ser-96 and tg33–His-95/WT prions were consistent with those observed after first isolation of the H95^+^ strain (24), demonstrating co-propagation of Wisc-1 and H95^+^ within hosts expressing WT–PrP^C^ (Fig. 11, *A–C*). Inoculation of Wisc-1 prions from tg33 mice into mice expressing Ser-96–PrP^C^ did not result in prion disease or neuropathology consistent with the H95^+^ strain, similar to the first passage of the WT/WT and Ser-96/WT CWD lineages (Fig. 11, *A–C*).

## Discussion

**Figure 12.**
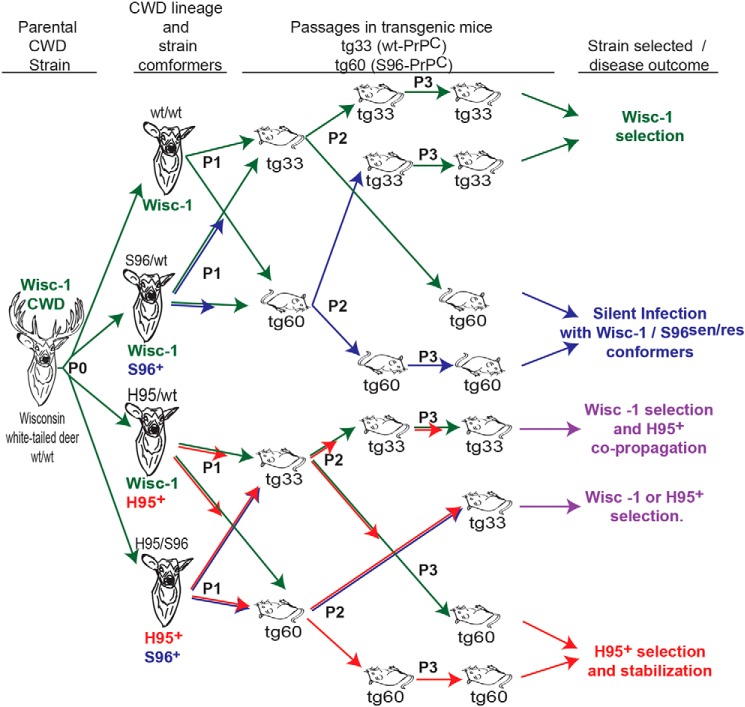
**Adaptive expansion of prion conformers in host environments that restrict prion propagation drives prion diversification and CWD strain emergence.** The transmission of Wisc-1 prions (*green arrows*) through white-tailed deer (*P0*) expressing permissive and restrictive PrP^C^ allelotypes and into transgenic mice expressing different PrP^C^ molecules (Gly-96 or Ser-96) resulted in co-propagation of conformationally heterogeneous prions associated with two biologically distinct CWD strains (Wisc-1 and H95^+^; *red arrows*) that differ in their transmission properties in tg60 mice (clinically affected mice depicted as inverted). Low-stability Ser-96–PrP^CWD^ conformers (S96^+^; *blue arrows*) failing to produce disease or adapt in tg60 mice after serial passage, however, produced disease consistent with Wisc-1 infection when inoculated in tg33 mice expressing G96-PrP^C^. The H95^+^ strain is adaptable in both tg lines and allelotypes (*i.e.* it can be encoded in WT, Ser-96, and His-95–PrP).

We show that prion strains are generated from the plasticity of the parental strain conformers during replication with nonhomologous PrP^C^ substrates arising from *PRNP* polymorphisms (P0). The iterative host selection (P1–P3) among these conformational species results in adaptation of new prion strains (Fig. 12). Using sensitive biophysical and *in vitro* replication tools, we show that distinct conformations of PrP^CWD^ co-exist in heterozygous deer, constituting an expansion of the conformational diversity compared with WT/WT deer infected with the same CWD isolate (Wisc-1).

PrP^CWD^ conformers encoding a novel CWD strain (H95^+^) emerged in cervid hosts expressing His-95–PrP^C^. The Wisc-1 and H95^+^ prion strain conformations are differentially reproduced in tg33 and tg60 mice. Other Wisc-1 conformers derived by nonhomologous conversion (S96^+^) were unstable and thus did not emerge during bioassay as distinct strains (*e.g.* those forming the PK-sensitive low-stability PrP^CWD^ multimers specific to Ser-96/WT and His-95/Ser-96 deer or S96^res^ types in asymptomatic tg60 mice). These S96 conformers may be adaptable (*i.e.* as a *bona fide* prion strain) in more compatible host PrP^C^ backgrounds compared with tg60 mice. Finally, our PrP^CWD^ measurements in brain from deer and transgenic mice demonstrate that Wisc-1 can interact with the most common deer PrP^C^ molecules with different efficiencies and disease outcomes.

We propose prion strain evolution occurs under the selective pressures that host PrP^C^ polymorphisms impose on the invading PrP^CWD^ strains, driving conformational diversification and emergence of new prion strains during adaptation to novel host–PrP^C^ environments. Nonhomologous PrP^C^ conversion limits the replication of founder prions and favors the expansion of arising PrP^CWD^ conformational diversity required for the emergence of new conformational “species.” The concept of host selection among multiple conformational variants co-existing in a single host has been previously described (9, 10, 32).

Transmission of Wisc-1 prions to heterozygous His-95 or Ser-96 deer limited conversion on WT–PrP^C^ as compared with WT/WT deer. The longest incubation period and the ∼6-fold lower levels of PrP^CWD^ in deer His-95/Ser-96 support this interpretation. Bioassay data and CDI measurements in Wisc-1–infected tg60 mice indicate a limiting role of Ser-96–PrP^C^ during *in vivo* prion infection. These bioassay results contrast with *in vitro* conversion assays, which showed Ser-96–PrP^C^ is misfolded into PrP^CWD^ as efficiently as WT–PrP^C^ (41). Conversely, when Ser-96/Ser-96 CWD prions were inoculated into tg33 (WT–PrP^C^) mice, the attack rate was low (19). The reduced *in vivo* PrP^CWD^ abundance indicates that His-95 and Ser-96 polymorphisms modulate various aspects of infection, including resistance to cellular clearance, neuroinvasion, neuropathology degree, and stable prion strain replication (15, 19–24, 42).

The His-95 and Ser-96–PrP^C^ may inhibit the oligomerization of WT–PrP^CWD^ or prevent their association with detergent-resistant membrane components, *e.g.* WT/WT prions were the most abundant at the top of the sucrose gradient. For other ruminant prions, rapid disease progression is associated with small PrP multimer size (43, 44). The shorter incubation period of Wisc-1 in WT/WT deer fits this association, supporting an oligomerization inhibitory effect for His-95 and Ser-96–PrP^C^ in heterozygous deer (15, 19). Dominant-negative inhibition in PrP^C^ heterozygous hosts can extend the incubation period (45, 46). Alternatively, the slower disease progression in heterozygous deer could be due to different strains competing for PrP^C^ (15). A similar effect occurs following co-infection with different prions (47) or during propagation of strain mixtures from field cases (48, 49).

Based on molecular weight, glycotype, and protease resistance, PrP^CWD^ generated in His-95/WT and Ser-96/WT deer is primarily Wisc-1–templated on WT–PrP. Overcoming the Ser-96 and His-95–PrP^C^ structural restrictions, however, has consequences on the conformational properties as indicated by different His-95/Ser-96 PrP^CWD^ glycotypes. In addition, most His-95 and Ser-96 PrP^CWD^ detected by CDI were protease-sensitive, suggesting these formed primarily PK-sen oligomeric structures. The endogenous N-terminal PrP cleavage patterns were distinct between CWD allotypes, suggesting that His-95 and/or Ser-96–PrP^CWD^ have different structural packing, and their N terminus is more exposed to host proteolysis than WT–PrP^CWD^. Alternatively, the novel C3–PrP fragment might represent amplification of an alternative conformation in His-95/Ser-96 deer (*i.e.* type-3 S96^res^). Endogenous N-terminal truncation of prions depends on the strain conformation (50–52).

The propensity of His-95/WT, Ser-96/WT, and His-95/Ser-96 prions to unfold with increasing GdnHCl (m) indicates novel PrP^CWD^ conformers were generated and/or amplified upon Wisc-1 conversion of nonhomologous PrP^C^. Prion strains can be differentiated by the structural stability and sedimentation properties of their corresponding conformers and multimeric states, even when co-existing in the same host (2–5, 32). The CSA monitors the transition from native aggregates to fully-denatured monomers of PrP^Sc^. In contrast, the WB-based techniques monitor either the partial solubilization of PrP^Sc^ (53) or conversion of rPrP^Sc^ to protease-sensitive oligomers (54) after exposure to denaturant. Thus, the data on the stability of soluble protease-sensitive oligomers and intermediates of PrP^Sc^ cannot be obtained with WB techniques and can lead to markedly underestimated values (35). The transitions from the native to the denatured state of cloned laboratory and human prions is sigmoidal indicating that one-step unfolding of closely-related conformers can be fitted by a standard sigmoidal transition model (4, 5, 29, 30, 37). The nonsigmoidal transitions we observed with His-95/Ser-96 and Ser-96/WT CWD prions also occur in human prions (3, 30) and in brain-derived β-amyloid (31). These two-step denaturation profiles of a mixture of conformers with distinct stabilities are direct evidence for a co-existence of distinct prions or prion-like amyloids (3, 30, 31). This would not appear to be a rare event; ∼40% of sporadic Creutzfeldt-Jakob disease cases involve two human prion strains in the same or different brain locations of the same patient (55–58). In addition to PrP^CWD^ conformational variability, the sedimentation profiles of WT–PrP^CWD^, as examined with 12B2-CDI, differed in the presence of His-95 or Ser-96–PrP^CWD^ suggesting these oligomeric assemblies generated WT–PrP^CWD^ with different properties compared with Wisc-1 in WT/WT deer. Analysis of His-95/WT with 8G8-CDI is indicative of a mixture of PrP^CWD^ structures with different amino acid composition.

The expansion of PrP^CWD^ diversity upon Wisc-1 nonhomologous replication in deer expressing His-95–PrP^C^ coincided with the accumulation of two strains (*i.e.* Wisc-1 and H95^+^) with distinct host range (24, 25). Importantly, and despite a loss of conformational diversity by serial passage in tg33 and tg60 mice, the strain-specific conformers of Wisc-1 and H95^+^ were differentially amplified depending on the recipient host PrP^C^. Even during nonoptimal replication conditions following first passage in tg33 mice, the H95^+^ was encoded in WT–PrP^CWD^ and could be reisolated in tg60 mice. Conversely, when tg60-adapted H95^+^ prions were transmitted in tg33 mice, H95^+^–specific PrP^CWD^ and deposition patterns were observed demonstrating the ability of H95^+^ to replicate using WT-, Ser-96-, and His-95–PrP^C^. Although certain prion strains show substrate PrP^C^ specificities, others are more promiscuous (*i.e.* classical BSE prions) (59–62).

Wisc-1 replication on Ser-96–PrP was unstable resulting in propagation of alternative S96^res^ conformers (type-2 and -3). These conformers were also unstable during *in vitro* conversion of Ser-96–PrP^C^ and, in the case of type-2, regenerated both the Wisc-1 (type-1) and type-3 PrP^CWD^ profiles. The 10-fold lower abundance of S96^res^ conformers in asymptomatic tg60 mice compared with symptomatic H95^+^-infected tg60 mice suggests their instability makes them less efficient converting Ser-96–PrP^C^, more prone to replication interference, and/or more susceptible to clearance. Whether S96^res^ PrP conformers can emerge as *bona fide* prion strains remains to be determined. Overall, Wisc-1 prions showed high replication affinity for WT–PrP^C^ and to a markedly lower extent for Ser-96–PrP^C^ molecules, whereas H95^+^ can readily replicate using the three different PrP^C^ allelotypes.

The contrasting host adaptability between the WT/WT and His-95/Ser-96 lineages supports the hypothesis that the origin of the H95^+^ strain is via conformational diversification of Wisc-1 during conversion of His-95–PrP^C^, instead of selection of a pre-existing H95^+^ conformer (*i.e.* encoded in WT–PrP^CWD^) from the original WT/WT isolate (Fig. 12). The lack of transmission of WT/WT, Ser-96/WT prions, and their P1 descendants into tg60 mice, as well as the absence of neuropathology resembling H95^+^, demonstrates that H95^+^ did not exist (as in a mixture) prior to passage of the Wisc-1 inocula into His-95–PrP^C^ polymorphic deer.

CWD is enzootic in various regions of the globe, and new cases are reported regularly, most recently in European cervids (63). Our data indicate the selective propagation environments established by host PrP^C^ polymorphisms drive and regulate the evolution and selection of CWD strains. Transmission studies of cervids with different PrP^C^ suggest a compatible interpretation (24, 64, 65). Our data may also provide an explanation for the lack of association between Met/Leu 132 PrP^C^ polymorphisms and CWD susceptibility in some affected elk populations (66). An increase in the Leu-132 PrP^C^ frequency may have favored unstable prion conversion with emergence and selection of a new strain that counteracted the protective effect of this polymorphism. We hypothesize the process of diversification of ecological roles during colonization of new environments by a replicating lineage, known as adaptive radiation, applies to the evolution of cervid prion strains.

Prions have been shown to respond to their changing environments (*i.e.* acquisition of drug resistance) (67), so we propose the adaptive diversification and selection of prion conformers could represent the mechanism behind this response. Finally, the observation of host (environment)-dependent expansion of conformational diversity, conformational speciation, and host strain selection reinforces the prion hypothesis by more intimately linking prion conformation to host resistance, strain emergence, and selection.

## Experimental procedures

Mouse studies were conducted in accordance with the Canadian Council on Animal Care Guidelines and Policies with approval from the Health Sciences Animal Care and Use Committee of the University of Alberta Animal Care and Use Committee (AUP 914). Deer tissues are stored in the bio-containment tissue archive at the Center for Prions and Protein Folding Diseases. These tissues were derived from a previous transmission study (15) performed at the University of Wisconsin, Madison, in accordance with the recommendations in the Guide for the Care and Use of Laboratory Animals of the National Institutes of Health and the School of Veterinary Medicine Animal Care and Use Committee at the University of Wisconsin (Permit No. V910). No external consent was required for the use of deer tissues.

### Experimental CWD prions

Brain homogenates from four different CWD PrP allotypes were obtained from orally-infected white-tailed deer (*O. virginianus*) and homogenized in phosphate-buffered saline solution as described previously (15). Brains from transgenic mice were homogenized to 10% (w/v) in sterile water using ceramic beads and a tissue homogenizer (Omni, Kennesaw, GA). All aliquots were stored at −80 °C until needed. Mice were inoculated intracerebrally with 30 μl of 10% (w/v) brain homogenate unless otherwise denoted (24).

### Enzymatic treatments

To assess for deer protease-resistant PrP^CWD^, 100 μg of total brain protein (final volume 70 μl) was treated with 150 μg/ml PK (Roche Applied Science) for 45 min at 37 °C. Detection of His-95/Ser-96 PK-res PrP^CWD^ required three times more total brain protein. Enzymatic digestions were terminated by heating in 2.5× Laemmli buffer (150 mm Tris-HCl, pH 6.8, 0.5% bromphenol blue, 25% glycerol, 5% (w/v) SDS, 12.5% β-mercaptoethanol) at 100 °C for 10 min. Protease digestion of tg33 and tg60 brain homogenates and PMCA reactions was performed using 80–100 μg of total brain–protein equivalents (BCA^TM^ protein assay, Thermo Fisher Scientific). Samples were treated with 50–100 μg/ml PK in a final volume of 70 μl of RIPA lysis buffer (1% Triton X-100, 1% sodium deoxycholate, 150 mm NaCl, 50 mm Tris-HCl, pH 7.4, 0.1% SDS, 1 mm EDTA). Detection of Ser-96 PrP-res from asymptomatic tg60 mouse was performed using three times more brain protein equivalents in parallel with a 10- and 100-fold dilution of H95^+^ and Wisc-1 controls from clinically affected tg60 and tg33 mice, respectively, diluted in tg60 normal brain homogenate and processed accordingly.

For de-glycosylation, total brain homogenate (25 μg of protein), in 0.7% SDS and 54 mm DTT, was denatured for 10 min at 100 °C. Samples were adjusted to 50 mm sodium phosphate, 1% Nonidet P-40 and deglycosylated with 1500 units of PNGase F (New England Biolabs) at 37 °C overnight. Reactions were terminated as described for PK digestions.

To compare the protease resistance of PrP^CWD^, 100 μg of total brain protein was incubated with 100 μg/ml proteinase K or thermolysin (at a final concentration of 1 μg/μl). Reactions were incubated at 37 °C (PK) and 70 °C (thermolysin). Aliquots were removed every 2 h for the first 12 h, and additional samples were taken at 24 and 48 h.

### SDS-PAGE and Western blotting

Protein concentration was determined using a micro-bicinchoninic acid assay kit (Thermo Fisher Scientific). Samples were denatured in 2.5× Laemmli buffer (150 mm Tris-HCl, pH 6.8, 0.5% bromphenol blue, 25% glycerol, 5% (w/v) SDS, 12.5% β-mercaptoethanol) at 100 °C for 10 min. Samples were resolved by SDS-PAGE, as described previously (24). Detection was performed using primary monoclonal antibodies: BAR224 (0.2 μg/ml diluted 1:10,000 in 5% (w/v) nonfat dry milk in TBST; Cayman Chemical); 8G8 (0.2 μg/ml diluted 1:2000; Cayman Chemical); 12B2 (0.2 μg/ml diluted 1:20,000 in TBST; Wageningen UR); 8H4 (0.2 μg/ml diluted 1:10,000; Abcam); Sha31 (0.2 μg/ml diluted: 30,000 in TBST; Cayman Chemical). Blots were developed using secondary horseradish peroxidase–conjugated or alkaline phosphatase–conjugated goat anti-mouse IgG antibody (Bio-Rad). Images were acquired on X-ray film (Super Rx; Fujifilm) or in a gel imager (Typhoon; Amersham Biosciences). The signal intensities of PK-res PrP and C-terminal PrP were measured in three independent experiments using ImageJ (National Institutes of Health) and compared by ANOVA using Prism 5.04 (GraphPad software).

### CDI

To investigate the prion spectrum in CWD, we applied CDI and CSA monitoring with europium-labeled antibody epitopes exposed in native α-helical state of normal PrP^C^ and hidden in the predominantly β-sheet structured prion state of prion protein (PrPSc) (2, 38, 39, 68, 69). Brain homogenates (5% w/v in 2% v/v Sarkosyl in PBS, pH 7.4) were divided into two aliquots: one untreated (native), and the other heated in GdnHCl (4 m final concentration) for 5 min at 80 °C (denatured). Both samples were then diluted 20-fold in H_2_O containing protease inhibitors (phenylmethylsulfonyl fluoride, aprotinin, and leupeptin at 4 μg/ml each) and 0.007% (v/v) of Patent Blue V (Sigma). Aliquots (20 μl) were loaded on 96-well Lumitrac 600 high-binding plates (E&K Scientific) prefilled with 200 μl of assay buffer (PerkinElmer Life Sciences). The plates were previously coated with mAb 8H4 (epitope 175–185) in 200 mm NaH_2_PO_4_ containing 0.03% (w/v) NaN, pH 7.5. Following sample addition, the plates were incubated for 2 h and then blocked with Tris-buffered saline (TBS), pH 7.8, containing 0.5% BSA (w/v) and 6% sorbitol (w/v) for 1 h at room temperature. The wells were then washed in TBS containing 0.05% (v/v) Tween 20. The captured PrP was detected with europium-conjugated 12B2 (amino acids 93–97 cervid numbering) or 8G8 (amino acids 100–105 cervid numbering). Plates were incubated at room temperature with the respective antibody for 2 h, washed with TBS-0.05% Tween 20, and developed with enhancement solution (Wallac Inc.). The time-resolved fluorescence (TRF) signal was measured with a multimode microplate reader (BMG LABTECH). The concentration of PrP was calculated from the CDI signal using a calibration curve prepared with either recombinant PrP(23–231) for samples containing full-length PrP^CWD^ or with recombinant PrP(90–231) for samples containing truncated PK-res PrP^CWD^. The TRF signal of the native sample corresponds to the 12B2 or 8G8 epitopes exposed in PrP^C^ but hidden in PrP^CWD^ and that are proportional to the concentration of PrP^C^ (3). The TRF signal of the denatured aliquot corresponds to the total PrP in the sample, and the concentration of PrP^CWD^ was calculated according to the following: [PrP^CWD^] = [PrP denatured] − [PrP native]. The concentration of PK-res PrP^CWD^ was calculated for samples treated with PK followed by denaturation in GdnHCl. The concentration of PK-sen PrP^CWD^ was calculated accordingly: [PK-sen PrP^CWD^] = [PrP^CWD^] − [PK-res PrP^CWD^].

### CSA

Assessment of structural stability was performed as described previously (3). Briefly, frozen brain homogenate aliquots were thawed and sonicated three times for 5 s at 60% amplitude with a sonicator 4000 (Qsonica), and the concentration was adjusted to ∼50 ng/ml PrP^CWD^. Aliquots were treated with increasing concentrations of 8 m GdnHCl containing 0.007% (v/v) Patent Blue V (Sigma) in 0.25 or 0.5 m increments. Samples were incubated for 30 min at room temperature and rapidly diluted with assay buffer (PerkinElmer Life Sciences) containing diminishing concentrations of 8 m GdnHCl to a final concentration of 0.411 m for all samples. Each aliquot was loaded into Lumitrac 600 high-binding plates coated with 8H4 antibody and developed according to the CDI method using antibodies 12B2 or 8G8 labeled with europium.

The TRF signal was converted into apparent fractional change of unfolding (*F*_app_) according to the equation, *F* = (TRF_OBS_ − TRF_N_)/(TRF_U_ − TRF_N_), where TRF_OBS_ is the observed TRF value, and TRF_N_ and TRF_U_ are the values for native and unfolded forms at a given concentration of GdnHCl. To establish the concentration of GdnHCl where half of the PrP^CWD^ is denatured (GdnHCl_1/2_), the data are fitted by a least-squares method with a sigmoidal transition model as shown in Equation 1.

(Eq. 1) $$F_{\text{app}}=F_{0}+\frac{\left(F_{\max}-F_{0}\right)}{1+e^{\left(\left(C_{1/2}-C\right)/r\right)}}$$

The apparent fractional change (*F*) in the TRF signal is the function of GdnHCl concentration (*c*); *c*_1/2_ is the concentration of GdnHCl, where 50% of PrP^CWD^ is dissociated/unfolded, and *r* is the slope constant.

The effect of protease treatment on the stability of PrP^CWD^ was established by subtraction of the fractional change values after PK treatment, from the *F*_app_ values obtained before PK treatment (Δ*F*_app_ = *F*^0^ − *F*^PK^). This value was the fitted with a Gaussian model to estimate the proportion and average stability of PK-sen PrP^CWD^ conformers, as shown in Equation 2,

(Eq. 2) $$\Delta F_{app}=F_{0}+A^{\left(-{\left(C-C_{0}\right)}^{2}\right)}$$

In this model, the PK-induced fractional change is Δ*F*; *F*_0_ is the fractional change at 0 m GdnHCl, and *c*_0_ is the GdnHCl concentration at the maximum height *A* of the peak.

### Sedimentation velocity of PrP^CWD^

Brain homogenates (10% w/v in PBS, pH 7.4, 2% Sarkosyl) from deer and tg mice infected with CWD were clarified by centrifugation at 500 × *g* during 5 min. Supernatants were layered on top of a 10–45% sucrose gradient prepared in PBS, pH 7.4, containing 1% Sarkosyl in thin wall polyallomer tubes (Beckman Instruments) and centrifuged at 50,000 rpm for 73 min at 5 °C in an Optima TL ultracentrifuge in an SW 55 Ti rotor (Beckman Instruments). Sucrose fractions were collected from the bottom of the tube and analyzed by CDI as described above. The excess of lipids and soluble proteins in fractions 9 and 10 precluded CDI measurements of total PrP^CWD^ with 8G8 (Figs. 4 and 10); the concentrations in those fractions are reported only for PK-resistant PrP^CWD^.

### Bioassays in transgenic mice and lesion profile analysis

Bioassays and lesion scoring were performed as described previously (24) in transgenic mouse lines expressing the deer *WT-PRNP* allele (tg33^+/+^ and tg33^+/−^ mice) or the *Ser-96–PRNP* allele (tg60 mice, which express 30% less PrP^C^ than tg33^+/+^ mice) (20). Titration experiments were performed as described previously (70). Animals were monitored for the appearance of clinical signs and disease progression. Individual incubation periods are expressed as dpi. The distribution of incubation periods was compared using Prism 5.04 (GraphPad software).

### PMCA

*In vitro* amplifications using WT or Ser-96–PrP^C^ substrates were performed as described previously (70) with modifications. Briefly, tg33 or tg60 normal brain homogenates were seeded with different dilutions of CWD prions and sonicated 30 s every 15 min during 24 h (1 round) or 48 h. PMCA reactions were supplemented with three Teflon® beads per reaction. Negative controls were seeded with brain homogenate from uninfected WT/WT white tailed deer. Nonsonicated controls were incubated at 37 °C. Normal brain homogenate substrate was not supplemented with saponin (70). PMCA was performed using a Q700 and Q700MPX sonicators (Qsonica) connected to circulating water baths (Thermo Fisher Scientific).

## Author contributions

C. D. V., J. G. S., J. M. A., and D. M. conceptualization; C. D. V., Chae Kim, T. H., and J. G. S. data curation; C. D. V., J. G. S., and D. M. formal analysis; C. D. V., Chae Kim, and T. H. investigation; C. D. V., Chae Kim, and T. H. visualization; C. D. V., Chae Kim, T. H., Chiye Kim, J. G. S., and D. M. methodology; C. D. V. and D. M. writing-original draft; C. D. V., A. H., J. M. A., J. G. S., and D. M. writing-review and editing; Chiye Kim and D. M. resources; J. M. A., J. G. S., and D. M. supervision; J. G. S. and D. M. project administration; C. D. V. performed experiments and results analysis; Chae Kim and T. H. performed CDI, CSA, and sedimentation velocity; Chiye Kim bred and genotype transgenic mice.

## Supplementary Material

Supporting Information

## Footnotes

2 The abbreviations used are:

PrP^Sc^: scrapie prion protein
PrP^C^: cellular prion protein
CWD: chronic wasting disease
PK: proteinase K
CDI: conformation-dependent immunoassay
CSA: conformation stability assay
GdnHCl: guanidine hydrochloride
PMCA: protein-misfolding cyclic amplification
res: resistant
PNGase: peptide:*N*-glycosidase
ANOVA: analysis of variance
tg: transgenic
PK-sen: protease-sensitive prion conformer
TRF: time-resolved fluorescence
dpi: days post-inoculation
WB: Western blotting.
