# Supplementary material for: Chronic wasting disease (CWD) prion strains evolve via adaptive diversification of conformers in hosts expressing prion protein polymorphisms
Source: J Biol Chem. 2020 Feb 28;295(15):4985–5001. doi: 10.1074/jbc.RA120.012546 (PMC7152757; doi:10.1074/jbc.RA120.012546)
Supplement: Supporting Information [file supp_295_15_4985__index.html]

Chronic wasting disease (CWD) prion strains evolve via adaptive diversification of conformers in hosts expressing prion protein polymorphisms. — CWD prion strain diversification in resistant cervid hosts — Chronic wasting disease (CWD) prion strains evolve via adaptive diversification of conformers in hosts expressing prion protein polymorphisms — CWD prion strain diversification in resistant cervid hosts — Supporting Information 

# Chronic wasting disease (CWD) prion strains evolve via adaptive diversification of conformers in hosts expressing prion protein polymorphisms

## Supporting Information

- Supporting Information (to be published online) - Figure S1
