# Supplementary material for: Chronic wasting disease (CWD) prion strains evolve via adaptive diversification of conformers in hosts expressing prion protein polymorphisms
Source: J Biol Chem. 2020 Feb 28;295(15):4985–5001. doi: 10.1074/jbc.RA120.012546 (PMC7152757; doi:10.1074/jbc.RA120.012546)
Supplement: Supporting Information [file supp_RA120.012546_158088_2_supp_483173_q6dp9v.pdf]

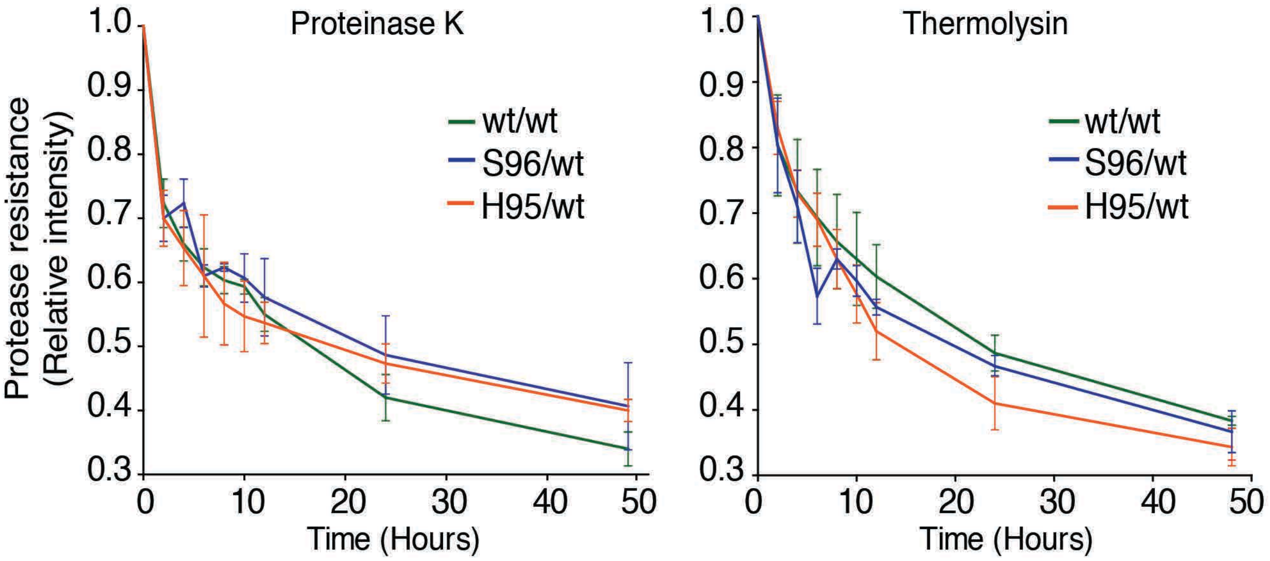

Figure S1. PrP^{CWD} protease resistance in heterozygous deer expressing wt-PrP^{C}. No significant differences were observed despite smaller levels of total PrP^{CWD} compared to wt/wt deer (Two-way ANOVA $p<0.05$; Tukeys multiple comparison post-test). Mean relative intensity and standard deviation indicated by bars. Densitometry was performed in three protease digestion experiments per CWD lineage.

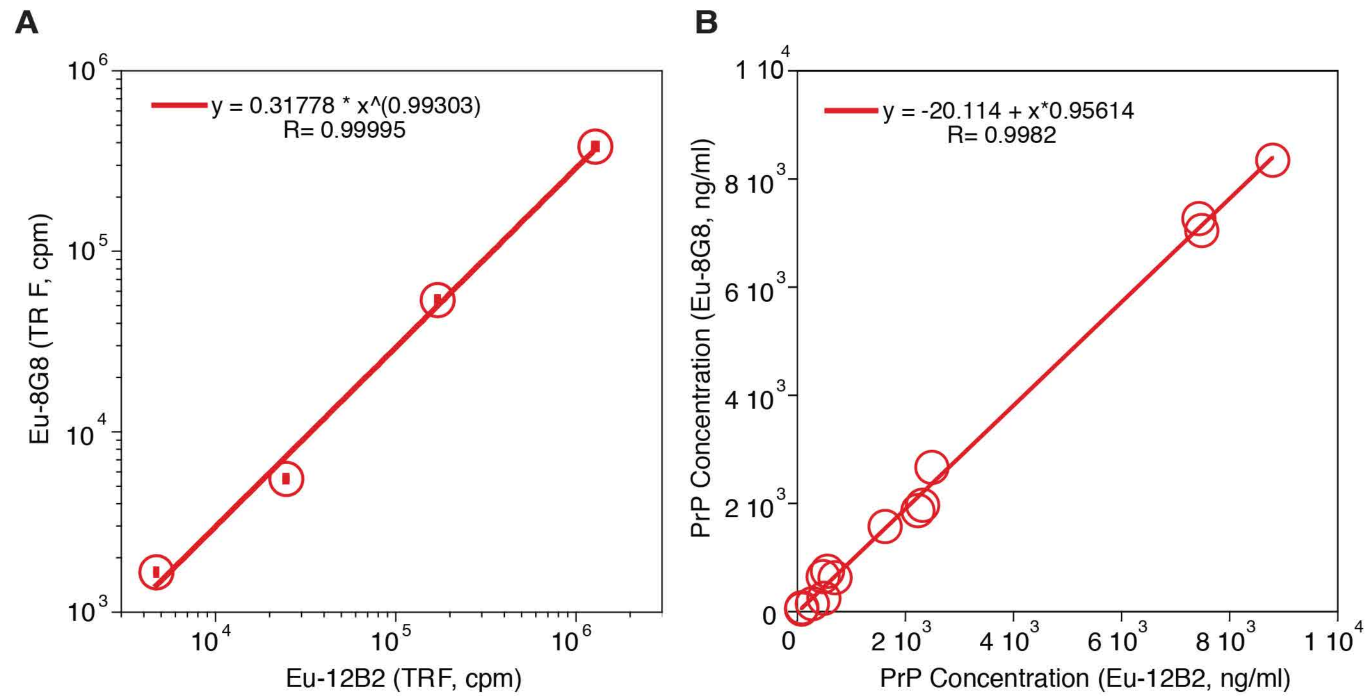

Figure S2. A) Calibration of CDI with Eu-12B2 and Eu-8G8 (epitope SQWNKP deer residues 100-105) with full recombinant PrP(23-231). B) Equivalent wt-PrP detection sensitivity with Europium-8G8 and Eu-12B2 in deer brain.

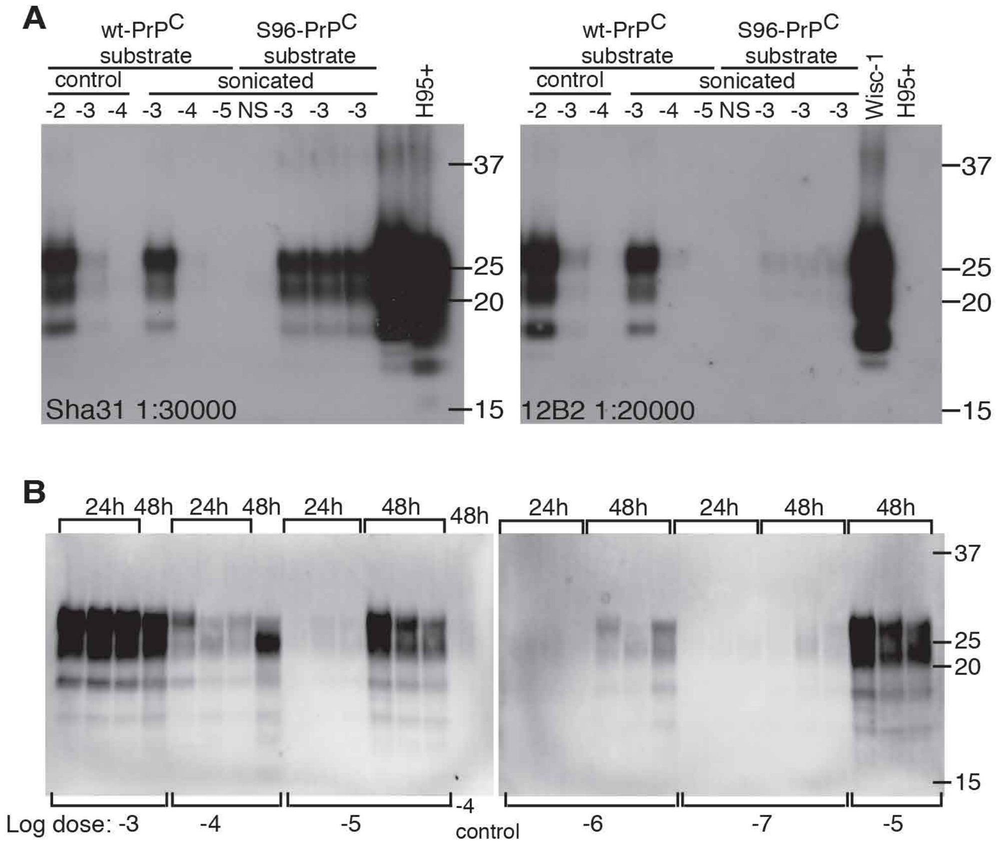

Figure S3. *In vitro* amplification of CWD lineages from deer and tg60 mice. **A**) PMCA propagation Wisc-1 from wt/wt deer with S96-PrP^{C} (mAbs indicated). **B**) PMCA detection of wt/wt prions on wt-PrP^{C} substrate after a single 48 hour round (Sha31 mAb 1:30000) - developed using gel imager. Unless otherwise specified, a single 24h round of PMCA was perfomed (30 seconds sonication at 60% amplitude every 15 minutes at 37°C). No sonication (control). No seed (NS).
